# Health care costs associated with gestational diabetes mellitus among high-risk women – results from a randomised trial

**DOI:** 10.1186/1471-2393-12-71

**Published:** 2012-07-24

**Authors:** Päivi Kolu, Jani Raitanen, Pekka Rissanen, Riitta Luoto

**Affiliations:** 1 UKK Institute for Health Promotion Research, Kaupinpuistonkatu 1, FI-33501, Tampere, Finland; 2 School of Health Sciences, University of Tampere, Tampere, Finland

**Keywords:** Cost, Gestational diabetes mellitus, Primary health care

## Abstract

**Background:**

The costs of gestational diabetes mellitus (GDM) screening have been frequently reported, but total GDM-related health care costs compared to the health care costs of women without GDM have not been reported. The aim of this study was to analyse GDM-related health care costs among women with an elevated risk of GDM.

**Methods:**

The study was based on a cluster-randomised GDM prevention trial (N = 848) carried out at maternity clinics, combined with data from the Finnish Medical Birth Register and Care Registers for Social Welfare and Health Care. Costs of outpatient visits to primary and secondary care, cost of inpatient hospital care before and after delivery, the use of insulin, delivery costs and babies’ stay in the neonatal intensive care unit were analysed. Women who developed GDM were compared to those who were not diagnosed with GDM.

**Results:**

Total mean health care costs adjusted for age, body mass index and education were 25.1% higher among women diagnosed with GDM (€6,432 vs. €5,143, p < 0.001) than among women without GDM. The cost of inpatient visits was 44% higher and neonatal intensive care unit use was 49% higher in the GDM group than among women without GDM. The delivery costs were the largest single component in both groups.

**Conclusions:**

A confirmed GDM diagnosis was associated with a significant increase in total health care costs. Effective lifestyle counselling by primary health care providers may offer a means of reducing the high costs of secondary care.

## Background

Gestational diabetes mellitus (GDM) is a term for diabetes first appearing during pregnancy and diagnosed with a glucose tolerance test [1]. The risk of GDM is associated with low physical activity, being overweight, and GDM in an earlier pregnancy [2-5]. In Finland the prevalence of GDM was 10.3–11.2% according to the Medical Birth Register for the period 2004–2006 [6]. GDM is a growing public health concern [7] and is associated with increased overall health care costs [8]. According to earlier research, women with GDM had 18% higher delivery stay costs than women without GDM [9]. In addition, antenatal outpatient costs due to visits to primary and secondary care were 25% higher among women with GDM than among women without a GDM diagnosis [10]. However, overweight (BMI ≥ 25) as a risk factor for GDM was associated with increased inpatient and outpatient visits during pregnancy [11,12].

GDM increases the postnatal health care burden due to an increased risk of neonatal complications, such as shoulder dystocia [13], foetal malformations [14] and, type 2 diabetes later in life [15,16]. There is an increasing trend in the incidence of GDM [7,17], which will consume more health care resources in the future. Thus cost analysis is a method of measuring the use of resources and describing the allocation of costs within the health care sector, and may help to create more cost-effective treatment procedures. To the best of our knowledge, there are no reports on the GDM risk group comparing the pre- and postnatal health care costs for women with GDM, and for women without a GDM diagnosis, but there are several studies related to the costs of screening [18-20]. We have previously reported the outcomes of GDM prevention in a cluster-randomised trial [21,22]. The aim of the original trial was to assess whether the occurrence of GDM can be prevented by dietary and physical activity counselling. As a result of the GDM prevention trial lower neonatal birthweight was seen in the intervention group compared to the group offered usual care, a favourable change in the consumption of fatty acids and saccharose intake in the intervention group. In addition, in the intervention group there was a smaller decrease in MET minutes per week for at least moderate intensity activity [22]. The aim of the present study was to describe GDM-associated health care costs between women with and without at least one GDM risk factor.

## Methods

This cost analysis was based on data from a cluster-randomised GDM prevention trial (N = 848), conducted at maternity clinics in Finland in the period from 2007 to 2009. During pregnant women’s routine visits to the maternity clinic, public health nurses recruited all women up to 12 weeks pregnant. The inclusion criteria of the original study were: BMI ≥ 25 kg/m², previous occurrence of GDM, or any signs of glucose intolerance, a macrosomic newborn (≥ 4500 g) in any earlier pregnancy, type 1 or 2 diabetes in first- or second-degree relatives or age ≥ 40 years. Women included in the trial were either primigravida or multigravida with singleton pregnancies. The exclusion criteria were: pathological value in baseline oral glucose tolerance test (OGTT) at 8–12 weeks’ gestation (blood glucose > 5.3 mmol/l at fasting, > 10.0 mmol/l at 1-hour or >8.6 mmol/l at 2-hour), type 1 or 2 diabetes before pregnancy, inadequate Finnish language proficiency, age < 18 years, twin pregnancy or physical limitation preventing physical activity.

In the present study, the case definition of GDM included women with a pathologic oral glucose tolerance test or ongoing insulin treatment. Women exhibiting no GDM diagnostic criteria formed a comparison group. The cost comparison included health care costs accruing to the municipality of residence and to the patient. The costs were evaluated from a societal perspective, including costs to the patient. This is because, in the tax-based health care system in Finland, the patients’ municipality of residence reimburses the real health care costs to the relevant hospital district. However, the daily charges for outpatient- and inpatient care also create a small cost to the patient. The cost analysis included all eligible women from the GDM prevention trial who had signed a study-participant consent form and had given us their permission to use their data in the Medical Birth Register and the Care Registers for Social Welfare and Health Care. Because the travel expenses and time costs related to the use of health services were assumed to be minor, they were not included in the calculation.

Costs were taken into account from the beginning of the pregnancy until the last day that the mother and her newborn spent in hospital after the birth. Information concerning medication and the number of visits to primary and secondary care was obtained from maternity cards filled in by the public health nurse at the maternity clinic. Information on visits to a diabetes nurse or a dietician was collected from questionnaires filled in by the mothers at the beginning of the study (at 8–13 gestational weeks) and again at 26–28 and at 37–39 gestational weeks. The information on the number of the mother’s inpatient hospital days before and after the standard delivery stay, the mode of delivery, the ICD-10 diagnosis code of the mother and newborn and the number of hospital days of the newborn were obtained from the Medical Birth Register and the Care Register.

Primary health care costs were based on the average national unit costs for health care, which includes visits to the public health nurse and to the doctor [23]. The costs of visits to secondary care, visits to a diabetes nurse and a dietician, the modes of delivery, inpatient days and neonatal intensive care units use were estimated by using the unit costs of the Tampere University Hospital, which was the hospital in which 93% of the deliveries took place and is the second largest hospital in Finland [24]. The unit costs were entered at the price level for 2009. Unit costs of obstetric outpatient and inpatient care included salary costs and administrative expenses, laboratory expenses as well as costs for all professionals participating in the provision of health care.

The cost evaluation included only inpatient days preceding and following delivery in hospital units related to pregnancy and GDM. The standard inpatient daily charge of 30 Euros was added to the unit cost, which was the cost to be paid by the patient. In the cost analysis, pre-delivery inpatient days were counted until the delivery and during the immediate post-partum period after delivery using the average national inpatient day cost [23]. Delivery cost and inpatient day costs due to delivery were counted separately using the Tampere University Hospital unit cost [24], which was used to obtain detailed DRG (Diagnosis Related Groups) information. Delivery unit costs included a standard number of inpatient days, depending on the mode of delivery. In addition, delivery-related operation costs included the salary costs of obstetric staff, including administrative expenses, medication and the cost of neonatal care in cases without an ICD-diagnosis. The newborn babies’ hospital stays were calculated separately in cases when the newborn needed care in a neonatal intensive care unit because of a disease involving organic complications or another ICD-10 diagnosis.

If the newborn had an ICD-10 diagnosis code, the costs of the newborn’s care were evaluated using the classification of the delivery hospital, where the costs of newborn babies were allocated to one of three possible unit cost categories, depending on the newborns’ birthweight, ICD-10 diagnosis code and the number of inpatient days in hospital. In the case of rooming-in, the costs of the newborn baby’s care were included in the mother’s delivery unit cost.

Medication costs included insulin costs but not the costs of glucose monitoring at home, as these were known to be minor [23]. Insulin costs were calculated for a period of 2.5 months and included health insurance reimbursements. According to the Finnish national guidelines, insulin treatment should be started, if necessary, at the 30th gestational week and should continue until delivery [25]. The costs of the oral glucose tolerance test (OGTT) as a diagnostic test for GDM were assumed to have been incurred once in both groups. In the original study, a pathologic OGTT result at 8–12 weeks’ gestation was a criterion for exclusion from the study, which meant that a large amount of information concerning the costs of OGTT was missed (N = 174).

### Statistical analysis

The association between the groups and continuous variables was tested with the Mann–Whitney U-test and with the chi-square test for categorical variables. Costs were reported as means and were rounded to whole Euros. Mann–Whitney U-test was used to analyse the differences between the two groups. Ratios or proportions of prenatal complications and mode of delivery variables were calculated using the chi-square test or, if the assumptions of the chi-square tests were not valid, Fisher's exact test. Total costs were adjusted for maternal age, body mass index and education. These adjustments were performed using ordinal regression analysis, because cost distributions were not normally distributed. The results were considered to be statistically significant if p < 0.05. All analyses were performed using SPSS software (version 19).

## Results

From a total of 848 women with an elevated risk of GDM, 251 (29.6%) had a confirmed GDM diagnosis by the end of the pregnancy, while 597 women (70.4%) formed a comparison group (Table 1). At the beginning of pregnancy the mean age of the GDM group was 30.6 years, and in the comparison group 29.2 years (p < 0.001). Women with a GDM diagnosis were significantly more overweight (28.8 vs. 24.9 kg/m²; p < 0.001), were more likely to have been diagnosed with GDM in earlier pregnancies (p < 0.001) and had a greater number of previous deliveries (p < 0.016). In contrast, there were no statistical correlations between a GDM diagnosis and the level of education or smoking habits. The sum of GDM risk factors was higher among women with GDM (1.51 vs. 0.92, p < 0.001) than among women without GDM (Table 1). There were no statistically significant differences between groups in prevalence of diseases such as elevated blood pressure, high blood cholesterol, diabetes, cardiovascular diseases, cancer, rheumatoid arthritis, bronchitis, depression or back problems (not shown in table).

**Table 1:** Characteristics (mean ± SD or frequency and percentage) and distribution of risk factors of GDM of women with and without GDM

	**Women with GDM**	**Missing**	**Comparison group†**	**Missing**	**p-value**
	*n = 251		**n = 597**		
Age categories					
−29	102 (40.6)		312 (52.3)		
30-34	90 (35.9)		200 (33.5)		
35+	59 (23.5)	0	85 (14.2)	0	0.001
BMI categories					
Normal (<25 kg/m²)	67 (27.0)		345 (57.8)		
Overweight (25 to 29.9 kg/m²)	91 (36.7)		175 (29.4)		
Obese (> = 30 kg/m²)	90 (36.3)	3	76 (12.8)	1	<0.001
Education level‡					
Low	98 (40.5)		192 (33.4)		
Medium	94 (38.8)		254 (44.3)		
High	50 (20.7)	9	128 (22.3)	23	0.15
Previous deliveries	1.10 ± 1.18	0	0.89 ± 0.99	0	0.016
Smoking during pregnancy					
No	225 (91.8)		556 (93.7)		
Stopped during first trimester	6 (2.4)		11 (1.9)		
Continued smoking after first trimester	14 (5.7)	6	26 (4.4)	4	0.60
Gestational diabetes in any earlier pregnancy	64 (28.3)	4	38 (7.3)	17	<0.001
Risk factors of GDM					
BMI ≥ 25 kg/m²	181 (73.3%)	4	247 (42.8%)	20	<0.001
Macrosomic children in earlier pregnancy	14 (5.7%)	4	12 (2.1%)	20	0.007
GDM in earlier pregnancy	64 (25.9%)	4	38 (6.6%)	20	<0.001
Diabetes in relatives	106 (42.9%)	4	227 (39.3%)	20	0.34
Age ≥ 40 years		4	7 (1.2%)	20	0.099
Polycystic ovarian syndrome	0	4	1 (0,2%)	20	1.00
Sum of risk factors	1.51 (0.74)	4	0.92 (0.75)	20	<0.001

*Inclusion criteria (one or more): pathologic oral glucose tolerance test, initiated insulin treatment during pregnancy, diagnosed GDM.

†Without GDM diagnostic criteria.

‡Low = vocational school or less.

Medium = polytechnic level.

High = academic education.

The adjusted mean total health care costs to the municipality and the patient were 25.1% higher among women diagnosed with GDM (€6,432 vs. €5,143, p < 0.001, Table 2). In addition, the use of insulin as a way to measure the severity of GDM was associated with higher total costs (€7,026 vs. €5,766, p < 0.001). The mean cost of visits to a public health nurse and to a doctor were slightly lower in the GDM group (€1,008 vs. €1,048, p = 0.019). The mean cost of visits to secondary care was 2.3 times higher for women diagnosed with GDM (€676 vs. €291, p < 0.001). Although the costs were minor, GDM was associated with increased costs (€40 vs. €1, p < 0.001) resulting from visits to a diabetes nurse, but there was no difference in the mean costs of visits to a dietician (p = 0.79). Insulin therapy was used by 29.1% of women diagnosed with GDM. The medication costs during the 2.5 month calculation period were €85, of which the proportion refunded by the Social Insurance institution of Finland was 42% of the total cost directly to the patient. The mean cost of total inpatient days before and after delivery was 44% higher in the GDM group (€491 vs. €341, p < 0.001) than in the comparison group.

**Table 2:** Use of health care services (mean and SD or frequency and percentage) and mean costs per person during pregnancy and postnatal period

		**Number of units**		Mean cost, €*		
	**Unit cost, €**	**Women with**	**Comparison**	**Women with**	**Comparison**	**p-value**
		**GDM (n = 251)**	**group (n = 597)**	**GDM (n = 251)**	**group (n = 597)**	
**Women**						
Visits to primary care	72 €/ visit	14.0 ± 3.3	14.6 ± 2.8	1,008	1,048	0.019
Visits to secondary care	208 €/ visit†	3.25 ± 2.9	1.40 +/− 1.6	676	291	<0.001
Visits to a diabetes nurse	91 €/ visit†	0.44 ± 0.86	0.01 ± 0.13	40	1	<0.001
Visits to a dietician	164 €/ visit†	0.01 ± 0.12	0.01 ± 0.14	2	2	0.79
OGTT	25 €/ test			25	25	
Insulin therapy	85 € / 2.5 month	73 (29.1%)	0 (0.0%)	25	-	<0.001
Hospital days before and after delivery	330 €/ day‡	1.49 ± 2.70	1.03 ± 2.20	491	341	<0.001
Delivery cost to the patient	30 €/ day	3.51 ± 1.22	3.37 ± 1.14	105	101	0.14
Delivery cost to the municipality (Table 3)				2,144	2,048	
**Newborn**						
Cost of neonatal intensive care unit to the patient§	30 €/ day	4.43 ± 1.57	4.41 ± 1.42	133	132	0.78
Cost of neonatal intensive care unit to the municipality (Table 3)				1,783	1,154	
Total mean costs, €				6,432	5,143	
Unadjusted						<0.001
Adjusted ∣∣						<0.001

*Costs are rounded to whole Euros.

†Including outpatient charge (27 € per visit).

‡ Including standard inpatient daily charge (30 € per day) and all costs to municipalities and patients.

§ Inpatient daily charge (30 € per day) during 1–7 hospital days.

∣∣Adjusted for maternal age, body mass index and education.

There were no statistical differences between the groups in terms of delivery cost to the patient (€105 vs. €101, p = 0.14). Also, the delivery cost to the municipality of residence was only slightly higher for women diagnosed with GDM than for women without GDM (€2,144 vs. €2,048, p = 0.051, Table 3). GDM was associated with a higher rate of labour induction (27.1% vs. 13.9%, p < 0.001, Table 4). The proportion of elective and emergency caesarean sections was higher in the GDM group (21.1% vs. 14.9%), whereas vaginal delivery was more frequent in women without a GDM diagnosis (78.9% vs. 85.1%, Table 4). As for the newborn baby, resuscitation was needed more often in the GDM group (5 vs. 1, p < 0.010). There were no statistical correlations between the groups in other prenatal complications or health outcomes (Table 4).

**Table 3:** Mean costs of delivery and treatment in a neonatal intensive care unit among women with risk of GDM

		**Women with GDM**		**Comparison group**		
	**Unit cost, €**	**Number of units**	**Cost, €**	**Number of units**	**Cost, €**	**p-value**
**Women**		n = 251		n = 597		
Vaginal delivery	1,758 €	178 (70.9%)	312,924	458 (76.7%)	805,164	
Instrumental delivery	2,477 €	20 (8.0%)	49,540	50 (8.4%)	123,850	
Elective cesarean section	2,741 €	20 (8.0%)	54,820	35 (5.9%)	95,935	
Emergency cesarean section	3,662 €	33 (13.1%)	120,846	54 (9.0%)	197,748	0.18
Mean delivery cost per person			2,144		2,048	0.051
Newborn*		n = 118		n = 183		
Neonatal intensive care unit †						
Category A	8,107 €/ period	3	24,321	9	72,963	
Category B	6,760 €/ period	5	33,800	0	-	
Category C	3,541 €/ period	110	389,510	174	616,134	
Mean newborn cost per person			1,783		1,154	p < 0.001

*Newborns without ICD-10 diagnosis were not included.

†Category A: Birthweight 1500–2499 g, inpatient days 1–22, including required examinations and treatments and ≤ 2 diseases with organic complications. Category B: Birthweight >2500 g, inpatient days 1–11, including required examinations and treatments and ≥ 1 disease with organic complications.

Category C: Birthweight >2500 g, inpatient days 1–7, including required examinations and treatments. No disease with organic complications.

**Table 4:** Mode of delivery and prenatal complications/ health outcomes

	**Women with GDM**	**Comparison group**	**p-value**
	**n = 251 (%)**	**n = 597 (%)**	
**Women**			
Induction of labour	68 (27.1%)	83 (13.9%)	<0.001
Mode of delivery			
Vaginal delivery	198 (78.9%)	508 (85.1%)	
Caesarean section	53 (21.1%)	89 (14.9%)	0.027
**Newborn**			
Serious perinatal complication	1	11	
Death	0 (0.0%)	0 (0.0%)	-
Shoulder dystocia	0 (0.0%)	3 (0.5%)	0.56
Bone fracture	1 (0.4%)	8 (1.3%)	0.22
Nerve palsy	0 (0.0%)	0 (0.0%)	-
Respiratory care	4 (1.6%)	4 (0.7%)	0.20
Blood change	0 (0.0%)	0 (0.0%)	-
Phototherapy	31 (12.4%)	56 (9.4%)	0.19
Resuscitation	5 (2.0%)	1 (0.2%)	0.010
Antibiotic treatment	25 (10.0%)	50 (8.4%)	0.46

Costs of outpatient visits both to primary and secondary care clinics were 28.6% higher, and inpatient service costs were 44% higher among women with GDM than among women without GDM (Table 5). The cost of delivery to the patient and the municipality was only slightly (4.7%) higher among women diagnosed with GDM, and was the largest single component of the mean costs in both groups. GDM was associated with 49% higher costs for treatment in a neonatal intensive care unit immediately after the birth than for the infants of mothers without GDM.

**Table 5:** Summary of allocation of mean costs to the patient and municipality among women with and without a GDM diagnosis and their newborn

	**Women with GDM**	**Comparison group**	**p-value**
	**n = 251**	**n = 597**	
	€	€	
**Women**			
Outpatient costs*	1,726	1,342	p < 0.001
Inpatient costs†	491	341	p < 0.001
Delivery costs‡	2,249	2,149	p = 0.054
Other costs§	50	25	
**Newborn**			
Neonatal intensive care unit costs‡	1,916	1,286	p < 0.001
Total costs	6,432	5,143	p < 0.001

*Sum of costs associated to visits to primary and secondary care.

†Sum of costs associated to inpatient days before and after delivery.

‡Sum of cost to the patient and the municipality.

§ Cost of oral glucose tolerance test (25 €) + medication.

## Discussion

A confirmed GDM diagnosis was associated with a significant increase in total health care costs. In addition to increased costs to the patient and to the municipality of residence, GDM was also associated with more frequent elective and emergency caesarean sections, which, in turn, is associated with an increased risk of adverse maternal outcomes [26]. On the other hand, the greater number of caesarean sections in the GDM group may explain the small number of delivery complications, such as bone fractures and shoulder dystocia occurring in the GDM group.

Visits to primary health care providers were not correlated with GDM, which is partly explained by the national guidelines concerning GDM. In Finland the municipalities provide antenatal care services for all pregnant women free of charge. Almost all pregnant women (99.7%) utilize municipal maternity care services, although there are also private services available [27], which minimized the amount of missing information. Women who are pregnant for the first time make a total of 14–18 visits to a public health nurse or a doctor in an antenatal clinic during their pregnancy [28]. The recommended number of visits to antenatal care providers for multiparous women is 10–14 [28]. However, GDM is associated with increased outpatient costs due to visits to secondary care, which was almost three times higher than the cost of a visit to a public health nurse or a doctor working in primary health care.

According to earlier studies being overweight, especially as a GDM risk factor, imposes a considerable and increasing burden on health services [11,12]. According to data from the Medical Birth Register for 2006 (N = 59,053), 34.2% of the women had at least one GDM risk factor and, of these, 20.4% had a confirmed GDM diagnosis [10]. According to our earlier study [10], the mean costs of primary health care visits, including the costs of OGTTs, were 40.5% higher among women with at least one GDM risk factor and a GDM diagnosis than among women without any risk factors or a GDM diagnosis. Even being moderately overweight before pregnancy correlated with higher hospitalisation costs [11]. In addition, total outpatient and inpatient costs were three times higher among the severely prepregnancy obese (BMI >35 kg/m²) than among women of normal weight (BMI <25 kg/m²) [11].

All participants in our study had a risk of GDM, which may increase pre-and postnatal health care costs. We have previously reported that the costs of visits to antenatal health care to see a doctor or a public health nurse did not differ according to the woman’s confirmed GDM status [10]. However, there were statistically significant differences between the numbers of visits and the costs of the group with neither GDM risk factors nor a GDM diagnosis and the group with at least one GDM risk factor and a confirmed GDM diagnosis [10].

This study had a number of limitations. For example, there was a risk of confounding which may reduce internal validity. However conditions such as elevated blood pressure were evenly distributed among groups which appears to offset this limitation. Another weakness was that the cost evaluation of inpatient days was based on inpatient days on wards related to pregnancy and gestational diabetes care. The use of ICD-10 diagnosis codes as inclusion criteria would have been more reliable for quantifying relevant inpatient days. On the other hand, GDM may increase the number of inpatient days for various reasons, which was a reason for the use of our current inclusion criteria. In addition, the study may underestimate the inpatient and outpatient days occurring after delivery, because readmissions were not taken into consideration. The costs were taken into account until the last day the mother or baby spent in hospital directly after birth, which was a precise cutpoint for cost evaluation.

Another potential limitation concerned unit costs, which were those of the Tampere University Hospital [24] and average national unit costs [23]. Health care costs were based on secondary care unit costs of one hospital district [24], which has to be taken into consideration when interpreting the study findings. However, the use of Tampere University Hospital unit costs provided an opportunity to evaluate secondary care costs in detail as the data available on national average health care costs did not include detailed delivery costs or costs of surgery based on ICD-10 diagnosis codes. The unit costs of the Tampere University Hospital [24] were similar to average national costs, partly because the Tampere University Hospital is a part of the Pirkanmaa Hospital District, which is the second biggest hospital district in Finland. Our study included only health care costs associated with GDM, but omitted economic costs such as the cost of productivity loss, which is a further limitation. Thus the total health care costs of gestational diabetes mellitus among high-risk women may have been underestimated. Since the data was based on a randomised intervention, the possibility that the intervention had an effect on GDM incidence cannot be denied. However, there were no differences between the trial groups in GDM incidence [21] and thus the intervention probably did not have an effect on the total cost of GDM.

## Conclusion

The study findings emphasize the potential importance of prevention programmes in saving money and improving the health of mothers and newborn babies. In further studies, it would be useful to evaluate the cost-effectiveness of health promotion programmes among GDM patients.

## Competing interests

The authors have no competing interests.

## Authors’ contributions

PK wrote the manuscript and researched the data, JR performed the statistical analysis. RL and PR reviewed and edited the manuscript and contributed to the discussion. All authors have approved the final form of the manuscript.

## Pre-publication history

The pre-publication history for this paper can be accessed here:

http://www.biomedcentral.com/1471-2393/12/71/prepub
